# Hippocampal Gene Expression Analysis Highlights *Ly6a/Sca-1* as Candidate Gene for Previously Mapped Novelty Induced Behaviors in Mice

**DOI:** 10.1371/journal.pone.0020716

**Published:** 2011-06-06

**Authors:** Simone de Jong, Martien J. H. Kas, Jeffrey Kiernan, Annetrude G. de Mooij-van Malsen, Hugo Oppelaar, Esther Janson, Igor Vukobradovic, Charles R. Farber, William L. Stanford, Roel A. Ophoff

**Affiliations:** 1 Department of Medical Genetics, University Medical Center Utrecht, Utrecht, The Netherlands; 2 Department of Neuroscience and Pharmacology, Rudolf Magnus Institute of Neuroscience, University Medical Center Utrecht, Utrecht, The Netherlands; 3 Institute of Biomaterials and Biomedical Engineering University of Toronto, Toronto, Ontario, Canada; 4 Department of Molecular Animal Physiology, Donders Institute for Brain, Cognition and Behaviour, Nijmegen Center for Neuroscience, Nijmegen, The Netherlands; 5 Centre for Modeling Human Disease, Samuel Lunenfeld Research Institute, Mount Sinai Hospital, Toronto, Ontario, Canada; 6 Department of Medicine, Department of Biochemistry and Molecular Genetics and Center for Public Health Genomics, University of Virginia, Charlottesville, Virginia, United States of America; 7 Department of Psychiatry, Rudolf Magnus Institute of Neuroscience, University Medical Center Utrecht, Utrecht, The Netherlands; 8 Center for Neurobehavioral Genetics, University of California Los Angeles, Los Angeles, California, United States of America; VIB & Katholieke Universiteit Leuven, Belgium

## Abstract

In this study, we show that the covariance between behavior and gene expression in the brain can help further unravel the determinants of neurobehavioral traits. Previously, a QTL for novelty induced motor activity levels was identified on murine chromosome 15 using consomic strains. With the goal of narrowing down the linked region and possibly identifying the gene underlying the quantitative trait, gene expression data from this F_2_-population was collected and used for expression QTL analysis. While genetic variation in these mice was limited to chromosome 15, eQTL analysis of gene expression showed strong *cis*-effects as well as *trans*-effects elsewhere in the genome. Using weighted gene co-expression network analysis, we were able to identify modules of co-expressed genes related to novelty induced motor activity levels. In eQTL analyses, the expression of *Ly6a* (a.k.a. *Sca-1*) was found to be *cis*-regulated by chromosome 15. *Ly6a* also surfaced in a group of genes resulting from the network analysis that was correlated with behavior. Behavioral analysis of *Ly6a* knock-out mice revealed reduced novelty induced motor activity levels when compared to wild type controls, confirming functional importance of *Ly6a* in this behavior, possibly through regulating other genes in a pathway. This study shows that gene expression profiling can be used to narrow down a previously identified behavioral QTL in mice, providing support for *Ly6a* as a candidate gene for functional involvement in novelty responsiveness.

## Introduction

With a prevalence of 10–20% worldwide, mood disorders affect a substantial number of people and finding the genetic risk factors will aid in prevention and treatment [1]. The heritability estimates for mood disorders range from 43% for panic disorder to 28% for anxiety disorder, indicating a genetic component to these disorders [2]. In animal research, behavior and novelty responsiveness are considered to be an important endophenotype in anxiety research [3], [4]. These behaviors are used to model different symptoms of mood disorders in mice, mainly fear, fatigue or loss of energy, and avoidance. These symptoms can be diminished when administering anxiolytic drugs [5], [6], [7]. Exploration behavior has been found to also be significantly heritable in mice [8].

Previously, a panel of mouse chromosome substitution strains (CSS) derived from host C57BL/6J and donor A/J mice [9], [10] was screened in several behavioral tests, including exposure to an open field arena and an automated home cage environment [3]. Subsequent fine-mapping in an F_2_-population revealed quantitative trait loci (QTL) for several novelty induced motor activity parameters on chromosome 15 [11]. The QTL region at mouse chromosome 15 has been implicated in these exploration behaviors before [12], [13].

The current study aims to explore the usefulness of genome-wide gene expression profiles for narrowing down quantitative trait loci (QTL) for behavioral parameters in mouse. Whole genome expression arrays were performed on hippocampal brain tissue of the same chromosome 15 F_2_ mouse population that was previously used for genetic mapping [11]. Because novelty induced locomotor activity is thought to reflect an endophenotpye for anxiety, the hippocampus was selected because of its role in emotion and cognition [14] and locomotor behavior in rodent species [15], [16].

Expression QTL (eQTL) analysis identified a number of *cis*- and *trans*-effects related to genetic variation at chromosome 15. In addition, we applied a weighted gene co-expression analysis (WGCNA) to discover networks of interconnected genes that are related to novelty induced motor activity levels [17], [18], [19], [20], [21]. Results from both the eQTL and network analysis indicated that genetic variation on chromosome 15 has genome-wide effects on expression. We also found that gene expression networks can be related to novelty induced motor activity parameters and can be used as tool in behavioral research. Most importantly, overlap between results from these methods revealed one gene, *Ly6a*, which we subsequently found to have a functional effect in gene knock-out mice. This study shows that gene expression data can be used to reveal relations between genomic variation, gene expression information, and behavioral parameters.

## Results

### Data preprocessing

**Figure 1:**
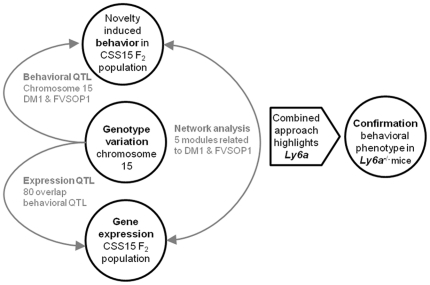
Overview of data analysis steps. This diagram shows the approach that was undertaken in this study in order to use gene expression data for fine mapping a behavioral QTL.

After quality control, expression arrays were background corrected, transformed and normalized according to the Lumi procedure [22]. Genes were then filtered based on detection values generated by Beadstudio©. We set the detection *p*-value at 0.01, leaving 13,450 of the 24,620 features on the microarrays for analysis. Figure 1 shows a diagram illustrating the approach of this study.

### Behavioral QTL regions

Behavior has been recorded in an automated home cage system, described before [11]. In short, the home cage environment is equipped with a home base shelter, a drinking spout, and two feeding platforms. On one feeding platform the mouse is exposed to the environment while the other platform allows sheltered feeding. The top unit contains an infrared camera and infrared light-emitting diode lights allowing continuous recording independent of lighting conditions in the test room.

**Figure 2:**
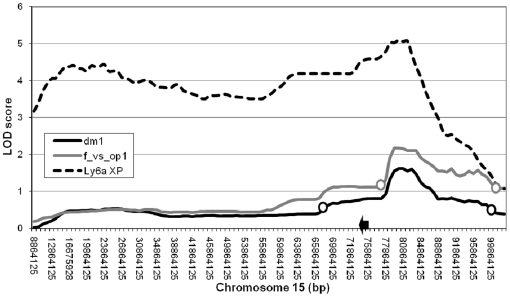
LOD scores of behavioral and expression QTLs. Plots of LOD scores across chromosome 15 (where markers available) are given for DM1 (distance moved on day 1) and FVSOP1 (frequency of visits to exposed platform on day 1), generated using a chromosome substitution strain F_2_-population. Open circles indicate the –LOD 1 support interval. In addition, the expression QTL is plotted for *Ly6a*, the only gene appearing in the results of both expression analyses. The black arrow indicates its location (74,825,308 bp).

Behavioral analysis was previously performed on 57 female CSS15F_2_ mice. A sex-specific effect of the A/J chromosome on the phenotypes of interest was observed and the behaviors were mapped for females only. In this study, a region for avoidance behavior parameters was mapped to chromosome 15 [11]. Since only one chromosome, i.e. chromosome 15, was interrogated, significance for the behavioral QTLs in a chromosome substitution strain was set at logarithm of odds (LOD)≥1.5 with a support interval of –LOD1 [11], [23]. The – LOD1 support interval of the parameter DM1 (distance moved on day 1) is 66,864,125 bp–98,864,125 bp, with the peak marker (rs13482668) at position 80,750,829 bp (mouse genome build 36.1) with a LOD score of 1.61. The interval for FVSOP1 (frequency of visiting exposed platform on day 1) is 76,492,248 bp–99,864,125 bp, peaking at the same marker with a maximum LOD score of 2.18. Plots are shown in Figure 2. The behavioral parameters are significantly correlated (*r* = 0.75, *p* = 9.2e-12).

### Expression QTL analysis

For expression QTL (eQTL) analysis, gene expression values are taken as phenotypes for genetic mapping. They were tested for association with genetic markers on chromosome 15, the only region variable between individual animals in our experiment. 1,000 random permutations resulted in a significance level of LOD≥3.22 for an estimated false discovery rate of 1%. This generated 136 significant eQTLs, of which 19 (14%) were located on chromosome 15. Of the top 10 probes (LOD≥4.93), 9 were located on chromosome 15. Multiple significant probes targeting genes on chromosome 15 were found to contain a SNP in the probe sequence (6 probes, of which 4 in top-10 results) and are likely to represent an artifact; these were removed from further analyses [24].

To identify possible candidate genes for the behaviors of interest, we looked at overlap between eQTLs and the region associated to behavior. 53 eQTLs (of which 10 target genes located on chromosome 15) overlap with both behavioral QTLs, while 27 more eQTLs (of which 3 target genes located on chromosome 15) only overlap with the broader peak of DM1. A list of significant eQTLs on chromosome 15 and their overlap with the behavioral QTL is given in Table 1. To confirm actual expression differences (*p*<0.01) between the parental strains A/J and C57BL/6J, we consulted another gene expression dataset that was available [24] *Card10* and *1700088E04Rik* are over-expressed in A/J compared to C57BL/6J. *Ly6a*, *Ly6e* and *Sub1* have lower gene expression levels in A/J than in C57BL/6J. Values for all the significant genes in the eQTL analysis can be found in Table S1.

**Table 1:** Genes on chromosome 15 resulting from eQTL analysis.

Illumina Probe ID	Max LOD score	Peak	Gene Symbol	Chr	Gene position (bp)	Accession	Overlap behavioral QTLs	Gene in QTL
1240142*	22.98	77864125	*Tomm22*	15	79501311	NM_199200.1	*	*
2260593*	12.33	79864125	*Mgat3*	15	80004151	NM_024177.2	*	*
3940324*	8.47	59864125	*Zfp706*	15	36930384	NM_028035.2	*	*
6520204	7.67	72864125	*Card10*	15	78605570	NM_172946.1	dm1	dm1/fvsop1
6650193	7.06	61864125	*Lrrc24*	15	76545706	NM_016905.1	dm1/fvsop1	dm1/fvsop1
5550400*	6.13	76338361	*2210021J22Rik*	15	85637419	NM_007554.1	*	*
1050288	5.4	85864125	*1700088E04Rik*	15	78965086	NM_013842.2	dm1/fvsop1	dm1/fvsop1
**6110605**	**5.1**	**80750829**	***Ly6a***	**15**	**74825308**	**NM_011631.1**	**dm1/fvsop1**	**dm1**
4780551	4.93	90251251	*Phf21b*	15	84615814	NM_009755.2	dm1/fvsop1	dm1/fvsop1
5050538	4.53	76338361	*Ly6e*	15	74786092	NM_133184.1	dm1/fvsop1	dm1/fvsop1
610546*	4.49	98864125	*Dbx2*	15	95453994	NM_172609.2	*	*
780463	4.48	55098638	*Sub1*	15	11913732	NM_010629.1	dm1	
5910735	4.07	95864125	*Grasp*	15	101054638	NM_017376.2	dm1/fvsop1	
1740195*	3.89	58864125	*Atf4*	15	80085929	NM_008612.1	*	*
460072	3.67	55098638	*Zfpm2*	15	40486588	NM_017465.1	dm1	
520524	3.54	86864125	*Maf1*	15	76181782	NM_026071.1	dm1/fvsop1	dm1/fvsop1
3120397	3.52	95864125	*Tef*	15	81632851	NM_025547.1	dm1/fvsop1	dm1/fvsop1
130592	3.32	64902010	*Adck5*	15	76406842	NM_024459.2	dm1/fvsop1	dm1/fvsop1
6290088	3.22	92864125	*Rbm9*	15	76912769	NM_178761.2	dm1/fvsop1	dm1/fvsop1

Expression QTL analysis of CSS15F_2_ mouse population yielded 19 results on chromosome 15. Maximum LOD score, the peak marker position and the –LOD 1 support interval is listed. In addition, we have indicated whether the eQTL and/or the gene itself was located in the behavioral QTLs. Asterisks indicate the probes of which the sequence contains a SNP polymorphic between A/J and C57BL/6J.

### Weighted gene co-expression analysis

#### Network construction and module detection

**Figure 3:**
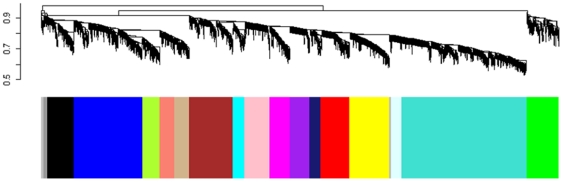
Network construction identifies distinct modules of co-expressed genes using hippocampus samples of CSS15F_2_ population (n = 37). The dendrogram was produced by average linkage hierarchical clustering of genes using topological overlap. Modules of co-expressed genes were assigned colors corresponding to the branches indicated by the horizontal bar beneath the dendrogram.

We used a network-based approach (weighted gene co-expression network analysis, WGCNA) to define clusters of co-expressed genes (‘modules’). It has been hypothesized that these modules represent biological meaningful networks. Once reconstructed, the correlation between each of the modules and the phenotype of interest is being examined. In other words, rather than generating a list of differentially expressed genes, this approach reconstructs networks of genes with related expression profiles that are thought to represent biological meaningful correlations. A detailed description can be found in the Methods section. In short, a weighted adjacency matrix containing pair-wise connection strengths was constructed by using the soft-thresholding approach (β = 7) on the matrix of pair-wise correlation coefficients. A connectivity measure (*k*) per gene was calculated by summing the connection strengths with other genes. Modules were defined as branches of a hierarchical clustering tree using a dissimilarity measure (1 - topological overlap matrix) [20], [25]. Each module (or branch) is subsequently assigned a unique color label which is visualized in the color band underneath the cluster tree (Figure 3). Our module detection method followed the standard WGCNA approach, which has been successfully used in multiple applications [18], [19], [20], [21], [26]. We identified 18 modules ranging in size from 25 genes in the Grey60 module to 868 in the Turquoise module.

#### Modules related to behavioral parameters

The next step was to identify the modules that are related to the behavioral measures of interest, DM1 (distance moved on day 1) and FVSOP1 (frequency of visiting exposed platform on day 1). When modules are related to these novelty induced motor activity phenotypes, the gene content can represent a biological pathway and harbor possible candidate genes.

To define a representative module expression profile (referred to as the module eigengene value), we summarized the (standardized) gene expression profiles of the module by their first principal component. The module eigengene can be considered a weighted (quantitative) average of the gene expression profiles in the module. The correlation between the module eigengene and the sample trait of interest is referred to as ‘eigengene significance’. A standard correlation test can be used to assess the statistical significance (*p*-value) of the eigengene significance.

Because DM1 and FVSOP1 both measure novelty induced motor activity levels, modules that overlap in these parameters were chosen as the focus of subsequent analyses. The Turquoise (868 probes; DM1 *r* = 0.36, *p* = 0.03; FVSOP1 *r* = 0.41, *p* = 0.01), Midnight blue (76 probes; DM1 *r* = 0.38, *p* = 0.02; FVSOP1 *r* = 0.46, *p* = 0.004), Lightcyan (72 probes; DM1 *r* = 0.36, *p* = 0.03; FVSOP1 *r* = 0.42, *p* = 0.01), Pink (177 probes; DM1 *r* = −0.35, *p* = 0.03; FVSOP1 *r* = −0.39, *p* = 0.02) and Purple (138 probes; DM1 *r* = −0.38, *p* = 0.02; FVSOP1 *r* = −0.45, *p* = 0.005) modules were shown to be most interesting for DM1 and FVSOP1. Figure S1 represents a heat map showing correlations and corresponding *p*-values for each module with DM1 and FVSOP1, as well as the genetic markers used. Values for all probes within the significant modules are listed in Table S2.

Genetic markers were most correlated with the Green (226 probes) Grey60 (25 probes), Black (184 probes), Blue (481 probes), and Grey module (30 probes). Only the Turquoise module had three probes containing a SNP in the sequence, constituting 0.35% of the total probes in that module.

To confirm actual expression differences between the parental mouse strains A/J and C57BL/6J, we consulted an independent gene expression dataset of hippocampal tissue which was available [24]. *Josd1* and *Ly6a* are under- and *Coq5* is over-expressed in A/J vs. C57BL/6J.

### Overlap of WGCNA and eQTL results

Of the 136 eQTLs, 28 fell within the modules resulting from the WGCNA. Of these, 26 were trans-regulated genes and they were found only in the Brown (1 probe), Grey60 (5 probes), Grey (4 probes), Blue (3 probes), Green (3 probes) and Black (13 probes) modules, which were shown to be associated to genetic markers in WGCNA. Two genes located on and controlled by chromosome 15 appeared in the modules: *Zfmp2* in Black and *Ly6a* in Turquoise. *Ly6a* is the only gene emerging in both lines of evidence related to our phenotype of interest. The expression QTL and location of the gene (74,828,318 bp–74,825,307 bp) is shown in Figure 2. The expression QTL of *Ly6a* covers a broad region but with a peak at the same location as the behavioral QT, rs13482668 (80,750,829 bp). At this location, expression of *Ly6a* shows an additive effect with higher expression for the C57BL/6J allele. The gene is located in the confidence interval of DM1 and at the border of that of FVSOP1. Limited genetic resolution interferes with precise indication of the QTLs, however, the results show that regulation of *Ly6a* expression peaks at the same genomic region as that of the behavioral QTL. *Ly6a* is found in the Turquoise module, which was found to be positively correlated to both behavioral parameters. The individual gene expression value of *Ly6a* was significantly positively correlated to both FVSOP1 (*r* = 0.44, *p* = 0.007) and DM1 (*r* = 0.34, *p* = 0.04). To exclude possible non-specificity of the probe sequence causing random hybridization signals, the probe sequence was blasted against the entire mouse genome (NCBI Blast; http://blast.ncbi.nlm.nih.gov/Blast.cgi). No non-specific binding sites were detected.

### Behavioral testing *Ly6a* ^−/−^ mice

In order to assess the functional relationship between novelty-induced motor activity levels and *Ly6a*, female *Ly6a* ^−/−^ mice (C57BL/6J background, n = 8) and C57BL/6J wildtype mice (n = 9) were tested using an automated open field system. Total distance moved (in cm) was used a measure of motor activity levels. Although it was impossible to test the mice in the exact same apparatus at this location, the novelty induced locomotor activity parameters in the home cage and the total distance moved in the open field arena both assess locomotor activity of the mice in the first hours they experience and explore their new environment.

**Figure 4:**
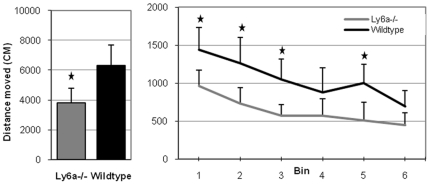
Behavioral analysis of *Ly6a* ^−/−^ mice confirms involvement in novelty induced phenotypes. *Ly6a* ^−/−^ (n = 8) and wildtype animals (n = 9) were tested in an automated open field system for 30 minutes. The data was analyzed using a repeated measures ANOVA for 6 bins of 5 minutes, after which individual bins were tested using independent *t*-tests (Bonferroni corrected). Total distance moved (in cm) is depicted in the bar diagram. The line graph shows the distance moved of *Ly6a* ^−/−^ and wildtype mice over the six bins. The error bars represent the standard deviation (SD). Stars indicate significance (Bonferroni corrected *p*<0.05).

A repeated measures ANOVA revealed a significant main effect of genotype on total distance moved in the six 5-minute bins (F(1,15) = 19.03, *p* = 0.001). Bins were tested separately using an independent sample *t*-test (Bonferroni corrected for multiple testing) revealing significantly lower activity of *Ly6a* ^−/−^ mice in bin 1 (t(15) = −3.83, *p* = 0.012), bin 2 (t(15) = −3.82, *p* = 0.012), bin 3 (t(15) = −4.14, *p* = 0.003) and bin 5 (t(15) = −4.11, *p* = 0.006). Data is shown in Figure 4.

The mice were tested again in the automated open field system in the dark phase. Again, the repeated measures ANOVA revealed a significant main effect of genotype on total distance moved on the six bins (F(1,15) = 15.01, *p* = 0.001) with lower activity for the *Ly6a^−/−^* mice. Independent sample *t*-tests (Bonferroni corrected for multiple testing) showed genotype differences for bin 2 (t(15) = −3.09, *p* = 0.042), bin 4 (t(15) = −3.59, *p* = 0.018), and bin 5 (t(15) = −3.11, *p* = 0.042).

To assess whether these differences are due to an effect of differences in motor function between wild type and knockout strains, a variable ‘velocity’ was computed by dividing the distance moved (in centimeters) by the duration (in seconds) over all 6 bins of 5 minutes in periphery and center. There was no significant difference in velocity between C57BL/6J and *Ly6a^−/−^* mice (t(15) = −1.11, *p* = 0.284). In addition, mice were subjected to a rotarod test (described in Methods section) [27]. The average latency to fall across three trials did not differ significantly between wildtype and *Ly6a^−/−^* mice (t(15) = −1.20, *p* = 0.25). Results are shown in Supporting Information S1.

## Discussion

The aim of this study was to use gene expression data to narrow down a QTL found to be associated to exploration and novelty-induced behavior. This behavioral QTL was mapped to chromosome 15 in a previous study, using a female F_2_-population of the chromosome substitution strain [11]. We generated whole genome expression data for this population and calculated expression QTLs to identify genes of which expression is regulated by the region of interest. Next, we applied a network based approach to define clusters of co-expressed genes (‘modules’) which are related to our phenotypes of interest. *Ly6a* (a.k.a. *Sca-1*) was found to be located on and regulated by the same region on chromosome 15 harboring the behavioral QTL. In addition, it belongs to a module correlated to novelty induced motor activity levels. These lines of evidence led us to test the exploration and novelty-induced behavior in *Ly6a* knock-out mice. The behavior was confirmed in this mouse knock-out strain, thereby providing support for *Ly6a* as candidate gene for functional involvement in novelty responsiveness.

This study yields insight in the relationships between genomic variation, gene expression, and behavior. By investigating a F_2_-population derived from a chromosome substitution strain, we know a priori that genetic variation that can be related to behavior and gene expression in this population can only stem from chromosome 15, since the rest of the genome is uniformly C57BL/6J.

Results from both the eQTL and weighted gene co-expression network analysis indicate that genetic variation at chromosome 15 has genome-wide effects on gene expression. The majority of eQTLs found were target genes located elsewhere (*trans*), although the *cis*-effects were relatively stronger. The number of *cis*-effects was comparable to those found on chromosome 15 in studies using recombinant inbred strains or outbred populations [28], [29]. The network analysis we carried out used only the most varying gene expression values to create a gene co-expression network. When examining results from the eQTL and network analyses, we find the genes that were shown to be regulated by chromosome 15 in the eQTL analysis were also found in the modules of the network analysis correlated to the genetic markers, thereby confirming their relation with this genomic location through separate statistical methods. In addition, we were able to link networks of co-expressed genes with novelty induced motor activity levels. This shows that gene expression can be used as a tool in behavioral research.

The overlap between genes found by these methods can relate genomic variation, expression information, and behavioral parameters. The only gene that was found to be regulated by chromosome 15 and identified in a module positively related to behavior of interest was *Ly6a*. This gene was also found to be significantly higher expressed in C57BL/6J than A/J hippocampus in an independent dataset.

*Ly6a* (lymphocyte antigen 6 complex, locus A) is an 18-kDa mouse glycosyl phosphatidylinositol-anchored cell surface protein of the *Ly6* gene family. This gene has been linked to stem/progenitor cell activity and cancer. The Ly6 proteins are thought to regulate or coativate cell signaling, although the exact mechanism by which they do so is unclear. For several members of the *Ly6* gene family, human orthologs have been identified. The syntenic region of *Ly6a*, along with other adjacent genes, is absent in rat [30]. There is also no human ortholog of *Ly6a*, yet other *Ly6* gene family members may function of *Ly6a* in a paralogous fashion. In addition, co-expression can indicate activation of genes belonging to a biological pathway. *Ly6a* could be affecting behavior indirectly through other genes in a pathway. Therefore, other genes in this module could be ‘guilty by association’ and have an effect on human behavior as well.

In order to functionally test the involvement of *Ly6a* in novelty induced motor activity levels, we examined behavior of the *Ly6a* knock-out strain. Thus far, the only phenotype studied in these mice was the altered proliferative response of their T-cells. The knock-out animals show a higher and more prolonged proliferation in response to T cell receptor-mediated activation, supporting a cell-signaling role for *Ly6a* [31]. No behavioral testing for the *Ly6a* knock-out strain had been described. The novelty-induced behavior was tested in an automated open field system. Although it was not possible to test the mice in the same home cage environment that was used before, the open field offers a close approximation of the behavior of interest and is generally used to assess novelty induced motor activity levels. In the original screen, distance moved in the open field arena (OF.DMA) was also assessed (data not shown). This parameter was positively, but not significantly, correlated to our parameters of interest (FVSOP1 & OF.DMA (*r* = 0.24, *p* = 0.07, DM1 & OF.DMA *r* = 0.14, *p* = 0.31) However, the correlation of OF.DMA with the expression of *Ly6a* is significant and shows the same directionality (OF.DMA & Ly6a *r* = 0.36, *p* = 0.03). In addition, in the original behavioral mapping, one of the peak markers of OF.DMA is the same as that of FVSOP1 and DM1 (rs13482612), containing *Ly6a*. The QTL pattern and thus possibly etiology of OF.DMA is related to FVSOP1 and DM1, but somewhat more complex and this might explain the lack of significance [11]. The *Ly6a* ^−/−^ mice showed significantly lower novelty induced ambulation in the open field recording when compared to C57BL/6J wildtype animals. This is consistent with our findings in the F_2_-population and therefore suggests that *Ly6a* is functionally involved in novelty induced motor activity levels. The C57BL/6J wildtype mice were not littermates of the *Ly6a* ^−/−^ mice, so separate breeding could have a confounding effect when comparing the two strains to each other. A trait-correlation of *Ly6a* expression was performed using the GeneNetwork website containing data of recombinant inbred strains (http://www.genenetwork.org, July 2010, sorted on correlation value). Phenotypes significantly associated with the expression of this gene contained three parameters related to novelty responsiveness in the top-6 parameters. Thus, even though the relation between the ontology of this gene in the light of novelty responsiveness related behaviors is unclear [30], the association appears to be consistent across independent datasets obtained from different mouse genetic backgrounds. Due to its involvement in stem cell activity [30], it is tempting to hypothesize that *Ly6a* is involved in hippocampal plasticity and influences novelty-induced behavior through this mechanism. In addition, it is possible that this gene exerts subtle influences on many other genes in its Turquoise module, or genes not detectable by microarray. Its gene family members, *Ly6e* and *Ly6c1*, appear in the results of eQTL and WGCNA analyses, respectively.

*Ly6a* is expressed in many tissues and it is involved in many receptor-ligand interactions. For example, it is expressed in muscle cells, where it interacts with caveolins, which in turn are implicated in forms of muscular dystrophy [32]. In addition, these mice show age-dependent signs of osteoporosis [33]. Although this effect appears in older mice (peaking at 7 months), we wanted to exclude possible motor defects by subjecting the mice to a rotarod test. The results show that the latency to fall does not differ between strains. Therefore, it is unlikely that a gross motor defect is responsible for the behavioral phenotype found in this study. Although the *Ly6a* ^−/−^ had a significantly higher bodyweight than the wildtype animals (t(15) = 2.88, *p* = 0.011) this was not correlated to latency to fall (*r* = −0.47, *p* = 0.055).

In conclusion, our results have revealed insights inter-relating genotype variation, gene expression, and behavioral data. We explored how to link genomic information, gene expression values, and behavioral parameters by combining statistical methods (i.e. eQTL and WGCNA). The combined approach highlighted the gene *Ly6a*. Subsequently, this gene was found to have a functional effect in the knock-out mouse. Although the association of *Ly6a* appears to be consistent, the ontology of this gene in light of novelty responsiveness related behaviors warrants further investigation.

## Materials and Methods

### CSS15F_2_ mice

We used a female F_2_-population of chromosome substitution strain 15 for this experiment as described elsewhere [11]. At least two weeks prior to the start of the experiment, the animals were moved from the stables to the adaptation room next to the experimental room. The animals were then 10–14 weeks old. They were tested consecutively in the open field, automated home cage environment, elevated plus maze, and light-dark box (fixed order), with a minimum of 1.5 weeks between tests. A description of these tests can be found in [11]. In short, the automated home cage environment is equipped with a home base shelter (in which mice mainly sleep during the light phase), a drinking spout, and two feeding platforms. On one feeding platform the mouse is exposed to the environment while the other platform allows sheltered feeding. The top unit contains an infrared camera and infrared light-emitting diode lights allowing continuous recording independent of lighting conditions in the test room. For the current study, the behavioral parameters of the automated home cage environment were further studied: distance moved on day 1 (DM1) and frequency of visiting the exposed platform on day 1 (FVSOP1). These parameters measure exploration during novelty and are assessed by means of motor activity levels (horizontal distance moved) during the first day of exposure to a novel environment. Genotypes were determined using SNPs and microsatellites as described before [11] Additional genotyping of two SNPs was performed in the CSS15F_2_ population in the current study. Primers were designed in ENSMBL to generate a PCR product. Sequencing was performed according to standard protocols on an ABI 3730 (Applied Biosystems) sequencer. A list of markers and positions can be found in Table S3.

### Dissection procedure

Mice were sacrificed at 3–4 months of age and their brains were quickly removed, frozen in liquid nitrogen, and stored at −80°C. After dissection this resulted in 37 hippocampal samples. The hippocampus has been linked to explorative behavior and is therefore a candidate region regarding our phenotypes of interest [34]. Brain samples (left hemisphere) were thawed from −80°C storage to −8°C in cryostat. Coronal sections of 300 µm thickness were taken. Frozen sections were laid down on a cooled steel plate, covered with parafilm and immediately covered with RNAlater (Ambion, #AM7024). Selected brain regions were punched out using a stainless steel punch needle (1 mm in diameter) filled with RNAlater connected to a syringe. Hippocampal tissue was taken in 6 sections starting at −2.06 mm Bregma, with two punches taken bilaterally in the first three sections, and three in the last three, aiming for 15 punches. These punches were pooled for each individual mouse prior to RNA isolation.

### RNA isolation and microarrays

RNAlater was pipetted off the samples. Punches were homogenized using disposable pestels (Fisher Scientific Pellet Stamp, #749521-1500). Phase separation was achieved using 750 µl TRIzol reagent (Invitrogen, #15596-018) and 200 µl chloroform (Merck, #8.222.65.1000), after which samples were precipitated in 500 µl isopropanol (Merck, #1.0934.2500). DNAse treatment (Qiagen Rnase-Free Dnase Set, #79254) was applied using the manufacturer's protocol, followed by an RNA clean-up procedure (Qiagen RNeasy MinElute columns, #74204). Samples were stored at −80°C. Total RNA concentration was determined using the Nanodrop ND-1000 and RNA quality was determined using Bioanalyzer RNA chips (Agilent Technologies RNA Nano kit, #5067-1511). Genome-wide RNA expression profiling was obtained with the Illumina MouseRef-8 V1.1 arrays using Illumina's standard protocol. In short, RNA samples were prepared with the Illumina TotalPrep kit amplification and labeling protocol (Ambion, #IL1791). Amplified and biotinylated CRNA was measured with a ribogreen assay (Invitrogen Quant-it™ Ribogreen, #R11490), and 750 ng of labeled cRNA was then used for array hybridization. BeadChips were scanned using an Illumina BeadArray reader. The raw microarray data is MIAME compliant and made available at gene expression omnibus (GEO) under accession number GSE29289.

### Statistical analysis gene expression data

We used Beadstudio© software version 3.2.3. to extract raw data and generate background, corrected gene-expression data. Further pre-processing was done using the Lumi package for R [22]. We applied a variance stabilizing transformation to preserve much of the gene expression variance and normalized data using the robust spline normalization method [35]. Chip quality and outlier detection was done by assessing quality statistics and plots before and after transformation and normalization. Both behavioral and expression QTL analysis were performed using the r/qtl package in R. We used the multiple imputation method, performing interval mapping (no SNP cofactors) and imputation of missing genotypes [36] and performed 1000 random permutations to determine the significance level for eQTL results. Significance for the behavioral QTLs in a chromosome substitution strain (LOD≥1.5) was set before [11].

Modules of co-expressed genes were identified using the weighted gene co-expression network analysis (WGCNA), developed by Zhang and Horvath [20], [21]. First, we constructed a correlation matrix for these genes. This matrix was then raised to a power (beta = 7 in this study) to achieve an adjacency matrix holding connection strengths. Connectivity is defined as the sum of connection strengths with the other network genes. With this, a topological overlap measure was calculated based on the number of shared neighbors. A hierarchical clustering tree of the genes was then constructed with these values, of which branches were cut with a dynamic tree cut algorithm to define the modules [37]. This method allows for testing the significance of the modules in the network to either a continuous or dichotomous outcome measure by assessing module eigengene significance. The module eigengene is the first principal component of a module and therefore represents a single gene expression value for all genes in a module per sample. The importance of genes can be assessed by looking at both gene significance and connectivity measures in the whole network and within modules. Analyses assessing polymorphisms between mouse inbred strains in probe sequences were performed *in silico* as described before [24].

### *Ly6a* ^−/−^ mice

*Ly6a* ^−/−^ mice on a C56BL/6J background were generated for previous studies [31], [38]. These mice were backcrossed to C57BL/6J for more than 10 generations and then intercrossed to establish +/+ and −/− mice. The *Ly6a* ^−/−^ mice were tested at a different location; the Stanford laboratory in Toronto, Canada. All procedures were approved by the animal care committee (ACC) at the Toronto Centre for Phenogenomics. Guidelines for ethical use of animals in research that TCP's ACC uses are provided by the Canadian Council on Animal Care (Protocol Permit # 2000-8138). The mice were group housed in a 12 hour light/12 hour dark cycle with *ad libitum* access to water and food. For the current study 8 *Ly6a^−/−^* and 9 wildtype C57BL/6J mice were tested, all female and between 4 and 4.5 months of age. All mice were test-naïve. At least two weeks prior to the start of the experiment, the animals were moved from the stables to the adaptation room next to the experimental room. They were tested the last two hours of their normal light/dark cycle in a fully automated open field system (Versamax Animal Activity Monitor, AccuScan Instruments). This uses horizontal and vertical x, y, and z sensors to detect the position and behavior of the animal in 5 minute bins for 30 minutes. Total activity in centimeters recorded in the cage was taken to represent total distance moved. Repeated measures ANOVA was used to asses main effects of genotype across bins, followed by independent *t*-tests (Bonferroni corrected) per bin. Mice were also subjected to a rotarod test (Economex Rota Rod, Columbus Instruments USA), testing their motor co-ordination and balance. Rotarod diameter was 4 centimeters and 4 rpm (rotations per minute) constant speed. The latency to fall in seconds was tested in three trials separated by 15 min inter-trial intervals. An independent *t*-test was used to assess significance.

## Supporting Information

Table S1 **Genes resulting from eQTL analysis.** Expression QTL analysis of CSS15F2 mouse population yields 19 results on chromosome 15. Maximum LOD score, the peak marker position and the –LOD1 support interval are listed. In addition, it is indicated whether the eQTL and/or the gene itself is located in the behavioral QTLs. Asterisks indicate probe of which the sequence contains a SNP polymorphic between A/J and C57BL/6J.
(XLS)

Table S2 **WGCNA results significant modules.** Weighted gene co-expression analysis of CSS15F2 mouse population yields five modules related to the phenotypic parameters of interest. Importance of genes in the whole network can be assessed by whole network connectivity. Hubs within modules are identified using the within-module connectivity measure. Asterisks indicate probe of which the sequence contains a SNP polymorphic between A/J and C57BL/6J.
(XLS)

Figure S1 **Heat map depicting module eigengene correlations and corresponding** ***p*** **-values.** Module eigengenes of the gene co-expression network are correlated with behavioral measures DM1 and FVSOP1 to identify modules of interest.
(EMF)

Table S3 **Genetic markers and their locations.**
(XLS)

Supporting Information S1 **Behavioral analysis Ly6a−/− mice.** Visual representations of behavioral recordings in an automated home cage (A) and rotarod (B) of *Ly6a* ^−/−^ mice and C57BL/6J wildtype mice.
(DOC)
