# Supplementary material for: Hippocampal Gene Expression Analysis Highlights Ly6a/Sca-1 as Candidate Gene for Previously Mapped Novelty Induced Behaviors in Mice
Source: PLoS One. 2011 Jun 6;6(6):e20716. doi: 10.1371/journal.pone.0020716 (PMC3108967; doi:10.1371/journal.pone.0020716)

**Supporting Information S1:** **Behavioral analysis Ly6a-/- mice**

**A) Ly6a-/- show behavioral phenotype in automated open field in dark.**


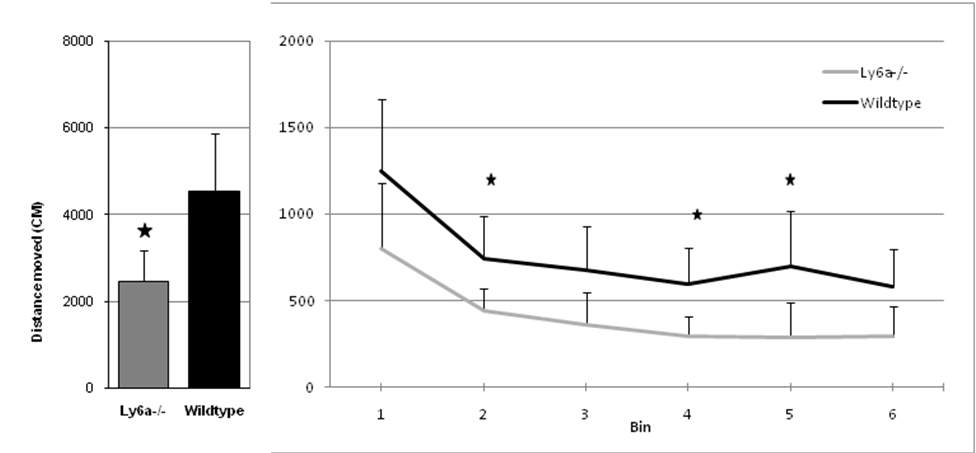


**B) Ly6a-/- show no gross motor defects in rotarod.**


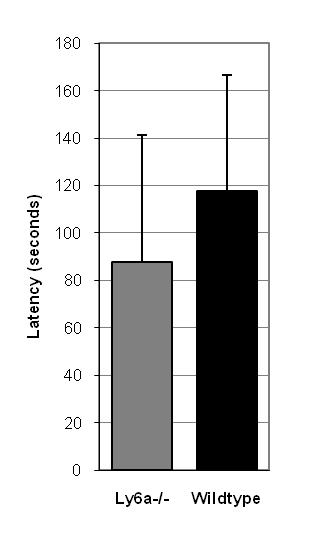

Supplement: Supporting Information S1 — Behavioral analysis Ly6a−/− mice. Visual representations of behavioral recordings in an automated home cage (A) and rotarod (B) of Ly6a −/− mice and C57BL/6J wildtype mice. (DOC) [file pone.0020716.s005.doc]
